# COVID-19 and pediatric inflammatory bowel disease: How to manage it?

**DOI:** 10.6061/clinics/2020/e1962

**Published:** 2020-05-25

**Authors:** Roberta Paranhos Fragoso, Maraci Rodrigues

**Affiliations:** I Centro de Ciencias da Saude, Universidade Federal do Espirito Santo, Vitoria, ES, BR; II Ambulatorio de Gastroenterologia Pediatrica, Prefeitura Municipal de Vitoria, Vitoria, ES, BR; III Departamento de Gastroenterologia, Hospital das Clinicas HCFMUSP, Faculdade de Medicina, Universidade de Sao Paulo, Sao Paulo, SP, BR

## Abstract

Pediatric gastroenterologists, family members, and caregivers of patients with inflammatory bowel disease (IBD) are on alert; they are all focused on implementing prophylactic measures to prevent infection by severe acute respiratory syndrome coronavirus 2, evaluating the risks in each patient, guiding them in their treatment, and keeping IBD in remission.

To face the current issues of the coronavirus disease pandemic, we have developed a synthesis of the main recommendations of the literature directed at pediatric gastroenterologists in control of patients with pediatric IBD and adapted to the national reality.

## INTRODUCTION

In January 2020, a new coronavirus was isolated in humans, and it was named the novel severe acute respiratory syndrome coronavirus 2 (SARS-CoV-2) after an outbreak of pneumonia of an unknown cause in late 2019 in the city of Wuhan, Hubei province, China (1).

The disease was called coronavirus disease (COVID-19), a disease with predominantly respiratory characteristics caused by SARS-CoV-2: a member of the beta coronaviruses originally described in bats, similar to the first human SARS coronavirus and the Middle East Respiratory Syndrome (1).

In March 2020, COVID-19 was declared a pandemic by the World Health Organization (WHO); it is threatening global health and destabilizing economies and societies worldwide (2).

## TRANSMISSION

SARS-CoV-2 has a high transmission rate, and its most common means of transmission include person-to-person, direct contact with contaminated secretions such as saliva droplets, sneezing, and coughing, and contact with contaminated objects or surfaces, followed by touching the mouth, nose, or eyes (3,4).

The virus was identified in nasopharyngeal and oropharyngeal swabs using real-time reverse transcription polymerase chain reaction (rRT-PCR) in the first cases of COVID-19 in the United States and in feces using the same method, which was probably the first identification of SARS-CoV-2 in the stool of a patient (5).

A published study demonstrated that there is a temporal difference in the appearance of a positive test depending on the origin of the samples collected (6). Fecal samples were positive 2-5 days after detection in respiratory specimens; additionally, they remained positive for an average of 11 days after respiratory samples were negative (7).

The viral load required for fecal-oral transmission is not known yet, but it does not depend on the presence of diarrhea (7).

The isolation of the virus in stool samples from patients with COVID-19 proved that SARS-CoV-2 could spread through feces. Based on its high viral infectivity, it was argued that exposure to an environment contaminated with feces, such as public toilets or areas with poor sanitation, could cause fecal-oral transmission when individuals touched their mouth, nose, or eyes with contaminated hands (8). Similarly, the virus could infect healthy family members of patients via the respiratory route through fecal aerosols caused by the use of toilet flush when sharing bathrooms (9). This type of transmission adds a major public health problem to the environment.

Transplacental or breast milk transmission has not yet been described; however, maternal-neonatal transmission shortly after birth has been reported (10).

## CLINICAL MANIFESTATIONS

Clinical manifestations of COVID-19 include the same symptoms as those found in other flu-like syndromes, for example, headache, fever, cough, coryza, odynophagia, myalgia, and anorexia (11).

Throughout the evolution of the pandemic and the increase in case descriptions, diarrhea, vomiting, and anorexia (not necessarily associated with respiratory symptoms), were observed in up to half of the cases of patients with COVID-19, and in their initial clinical presentations (12,13).

Reports of anosmia are frequent and more specific during SARS-CoV-2 infection (14).

Most cases of SARS-CoV-2 infection are mild with upper respiratory symptoms (pharyngeal congestion, sore throat, and fever) for a short period or even asymptomatic (15).

Moderate illness can manifest with mild pneumonia and be accompanied by flu-like symptoms without complications or manifestations related to a serious condition (15).

As COVID-19 progresses to a severe form, in addition to the flu-like symptoms, there is the occurrence of some of the following manifestations and laboratory findings: tachypnea, hypoxia, decreased consciousness, depression, coma, convulsion, dehydration, difficulty with eating, gastrointestinal dysfunction, myocardial injury, elevation of liver enzymes, coagulation dysfunction, rhabdomyolysis, and injury to vital organs. A critical outcome with rapid disease progression results in the need for support in an intensive care unit (15).

Although fever is a warning sign in this pandemic time of COVID-19, it is not always present in children (15).

Fortunately, children have a predominantly benign course, with mild symptoms or even no symptoms, and there is almost no reported mortality (16,17). However, children may play a major role in community-based viral transmission, especially in the home environment (11). The better evolution of COVID-19 in children is due to a weaker immune response (11).

Some hypotheses have been discussed to explain the better clinical evolution of COVID-19 in children, such as the lower systemic inflammatory response and the release of pro-inflammatory cytokines; as well as the lower virus concentration in the respiratory tract, probably related to the difference in the expression of angiotensin-converting enzyme receptor 2 in the respiratory tract of children than that of adults (16,17).

## DIAGNOSIS

According to the Guidelines for the Diagnosis and Treatment of COVID-19 promulgated by the Health Ministry of Brazil (published on April 6, 2020), the diagnosis of COVID-19 depends on clinical-epidemiological investigation and physical examination (18). The Health Ministry recommends that the laboratory diagnosis of COVID-19 be based on the rRT-PCR test, which amplifies the RNA sequence of the virus, enabling its identification. The most frequently used samples are the nasopharyngeal or oropharyngeal swab (18). Serological tests for identifying IgM and IgG antibodies against SARS-CoV-2, which are performed as rapid tests, are not recommended for the diagnostic confirmation of patients with symptoms of recent onset, although they have good diagnostic accuracy in patients with clinical manifestations lasting more than 8 days (18).

The WHO recommends the following combinations of laboratory tests for the diagnostic confirmation of SARS-CoV-2 (19):

- Confirmed positive PCR for the SARS-CoV-2:
  1. at least 2 different clinical specimens (nasopharyngeal and stool)
  2. or the same clinical specimen collected on 2 or more days during the course of the disease (2 or more nasopharyngeal aspirates)
  3. or 2 different assays or repeated PCR using the original clinical sample on each occasion of testing
- Seroconversion by enzyme-linked immunosorbent assay or immunofluorescence antibody:
  1. negative antibody test on acute serum followed by positive antibody test on convalescent serum
  2. or a four-fold or greater rise in antibody titer between acute and convalescent phase tested in parallel

The sensitivity of the PCR tests for SARS-CoV-2 depends on the specimen and the time of testing during the course of the illness.

It is recommended to obtain a chest X-ray for all patients with suspected pneumonia. Pediatric patients with COVID-19 who underwent chest tomography showed fewer changes and more concentrated lesions in the bronchial walls compared with adults (20).

However, it is necessary to assess the need for tomography on a case-by-case basis, especially due to the risk of early exposure of the child to ionizing radiation.

### What is important to know for the care of patients diagnosed with pediatric IBD during the COVID-19 pandemic?

Consider that IBD in children tends to be more extensive and severe than in adults, with a consistently greater need for immunomodulators and biological agents for them to remain in remission (21). Despite this, the first cases of pediatric IBD and SARS-CoV-2 infection described in an international database for the epidemiological survey on COVID-19 in children and adults, SECURE-IBD (Surveillance Epidemiology of Coronavirus Under Research Exclusion) did not show an unfavorable evolution for children (22).

It was proposed that the use of immunomodulators in the treatment of IBD patients may block the effects of the cytokine storm on the inflammatory response of these patients, leading to the control of inflammation of the bowel mucosa and prevention of COVID-19 pneumonia (23).

Data collected from the SECURE-IBD platform (22) described 704 patients, 9 cases in Brazil until April 22, 2020. There were cases described (3 cases of children between the ages of 0-9 years and 28 cases between 10-19 years old) of IBD associated with COVID-19, but no cases of ICU admission or death, although 11% of the 10-19 age group required hospitalization.

In pediatric care, family members and IBD patients have asked about the risks of SARS-CoV-2 infection, what precautions to take, and especially how to maintain IBD treatment, particularly those patients using immunosuppression. Therefore, in this paper, the implications of SARS-CoV-2 infection on patients undergoing pediatric IBD treatment are presented.

### 1. What are the general procedures and precautions to be taken during the COVID-19 pandemic in patients with pediatric IBD?

Based on the data available in the literature, individuals with IBD, with or without immunosuppressive therapy and a biological agent, appear to be at no greater risk of contracting SARS-CoV-2 infection when compared to the general population (24). Possibly, factors such as the better adherence of these pediatric patients to protection measures, supervised by their families, such as hygiene care and social distance, corroborate this report. In addition, the pediatric population is less affected by COVID-19 (16).

**Figure 1:**
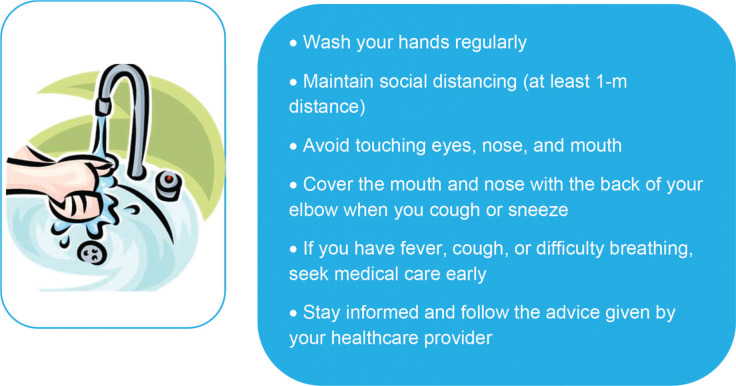
General measures and precautions to be taken during the COVID-19 pandemic in pediatric IBD. Modified from Danese et al. (25) and Turner et al. (26).

The procedures to protect and control the spread of the coronavirus can be summarized (25,26) as described below (Figure 1):

- Frequent handwashing, with soap and water for at least 20 seconds, especially in a public environment or after coughing, blowing the nose or sneezing, or after using the toilet
- If soap and water are not available, hand sanitizer with a minimum alcohol concentration of 60% may be used, with attention to cleaning all surfaces of the hands. Attention should be paid to the use of hand sanitizer, especially to the risk of domestic accidents, such as burns in children.
- Avoid touching the eyes, nose, or mouth until hands are washed.
- Cover the mouth and nose with the back of the elbow when sneezing or coughing.
- Practice sanitary hygiene and proper disposal of waste.
- With infants, it is necessary to be careful while changing diapers, properly storing them in waste containers, and ensuring that they are kept tightly closed.
- Thoroughly launder clothing used outside the home (in an emergency consultation, going to the clinic or hospital).
- Cleanse and disinfect surfaces that are touched daily, including toys, especially if there are flu-like symptoms.
- In children older than 2 years and adolescents with flu-like symptoms or infected with SARS-CoV-2, the use of a mask is recommended to reduce viral spread.
- In uninfected children and adolescents, masks should be worn when away from home.
- Avoid close contact with people affected by COVID-19.
- Maintain a social distance of 1 to 2 meters in public locations.

Children need to have the supervision of a caregiver and playful stimulus to follow the hygiene guidelines and incorporate these procedures into their daily lives. Shared bathrooms need cleaning and proper garbage disposal, and small children should not be allowed to access them unaccompanied.

### 2. How can I keep consultations for pediatric IBD during the COVID-19 pandemic?

**Figure 2:**
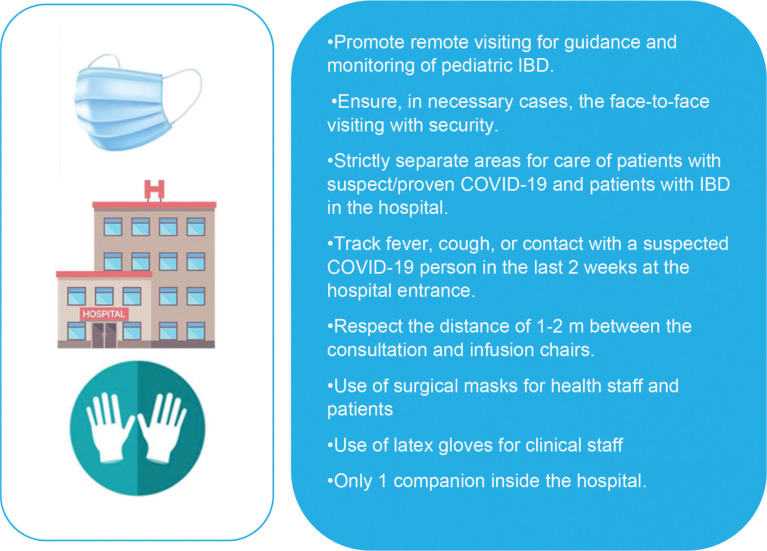
Remote and face-to-face monitoring of pediatric IBD during the pandemic COVID-19. Modified by Danese et al. (25), Turner et al. (26), and Fiorino et al. (27).

Face-to-face consultations can be changed to remote visits (phone, email, video call), reducing patient attendance at the hospital if not strictly necessary. Exceptions are a relapse of the disease, exam collection, or infusions. In such cases, there should be restricted areas for the care of patients with suspected or diagnosed COVID-19, separate from children with IBD without SARS-CoV-2 infection (Figure 2). The use of surgical masks for the health team and for all patients while they are in the hospital environment should be maintained (27).

**Figure 3:**
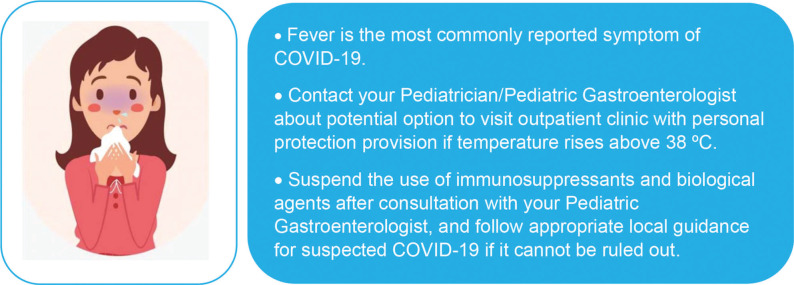
Recommendations for pediatric IBD patients with fever during the COVID-19 pandemic. Modified by Mao et al. (29).

- In all remote consultations, the medical record must be registered.
- In remote consultation of a stable patient, global guidance is recommended. Check the general condition of the patient, the proper use of the prescribed medication, and the maintenance of the treatment. Conduct an epidemiological investigation for COVID-19 in all consultations. In addition to the general impression of the disease reported by the patient and/or their family, the patient should perform, if necessary, inflammatory control tests (C Reactive Protein and fecal calprotectin) (27).
- In remote consultations, uninfected children with IBD should continue immunosuppressive medications and/or biological therapy. In cases of acute treatment, face-to-face assistance is recommended. There is no consensus regarding the exchange of medications or optimization of those being used during the pandemic, and therefore, the determinations must be individualized in each pediatric gastroenterology service and the decisions shared with the family.
- In the case of an asymptomatic patient using immunomodulators and a history of home contact with COVID-19, the rRT-PCR test for SARS-CoV-2 should be requested. If the test is positive, the medication should be suspended for two weeks and the immunosuppressive treatment should only be resumed after two negative tests (28).
- In the remote consultation of a patient with IBD and flu-like symptoms, it is advised that, in case of fever, cough, and difficulty breathing, the pediatrician/pediatric gastroenterologist should be immediately consulted for face-to-face evaluation.
- In face-to-face consultations, for patients with fever or suspected COVID-19, immunosuppressants should be suspended and local consensus for SARS-CoV-2 infection should be followed (figure 3). The treatment can be continued after the complete resolution of symptoms or ideally after performing 2 nasopharyngeal swab-negative rRT-PCR tests, collected with an interval of more than 24 hours (28) (Figure 3).
- Sometimes hospitalization may be necessary, due to severe IBD relapse and more rarely by association with SARS-CoV-2. COVID-19 does not appear to be a risk factor for relapses in IBD (29,30).
- Children admitted to the hospital during the pandemic are allowed to have a companion, but without replacement during the entire hospital stay.
- During the COVID-19 pandemic, elective endoscopic examinations should be postponed. The performance of upper gastrointestinal endoscopy and ileocolonoscopy is indicated only for potential cases (Cytomegalovirus research) and emergencies. In exceptional situations, when it is not possible to postpone the endoscopic procedure, it should be performed respecting the recommendations published by the Brazilian Society of Digestive Endoscopy (31).
- Other actions implemented in the various national centers include facilitating the administration of medicines to patients (for example, changing the hours of operation of the pharmacy and increasing the number of doses dispensed). In the case of infusions in a hospital during the pandemic, it is recommended to pre-screen the symptoms and temperatures, limit or reduce the number of patients accessing the infusion room, use masks and gloves (patients and assistant staff), and maintain appropriate space between infusion chairs.

In all remote and face-to-face consultations, recommendations on the prevention of exposure and the practice of social isolation should be given.

### 3. Can vaccines be administered to pediatric IBD patients during the COVID-19 pandemic?

**Figure 4:**
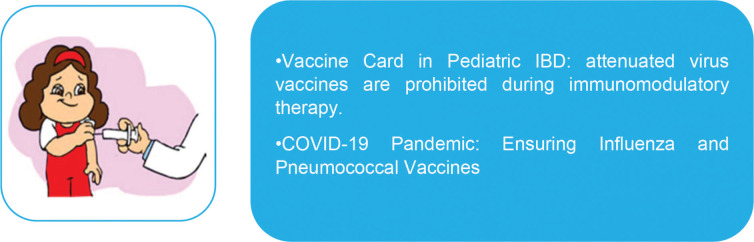
Recommendations on vaccines for pediatric IBD during the COVID 19 pandemic. Modified by Dipasquale & Romano (32).

- It is important to keep the vaccination card up to date for all children and adolescents with IBD. During the COVID-19 pandemic, it is necessary to make sure, mainly, that vaccines for Influenza and Pneumococcus are carried out, with the aim of avoiding respiratory complications that could confuse the diagnosis of COVID-19 (32) (Figure 4).

### 4. What are the specific procedures in relation to each of the medications used by patients with pediatric IBD during the COVID-19 pandemic?

According to the European Society of Pediatric Gastroenterology Hepatology and Nutrition (ESPGHAN), preliminary data from IBD in pediatric patients during the COVID-19 pandemic support the maintenance of standard IBD treatment, including the use of biological agents, especially in patients with a more serious disease (26).

**Figure 5:**
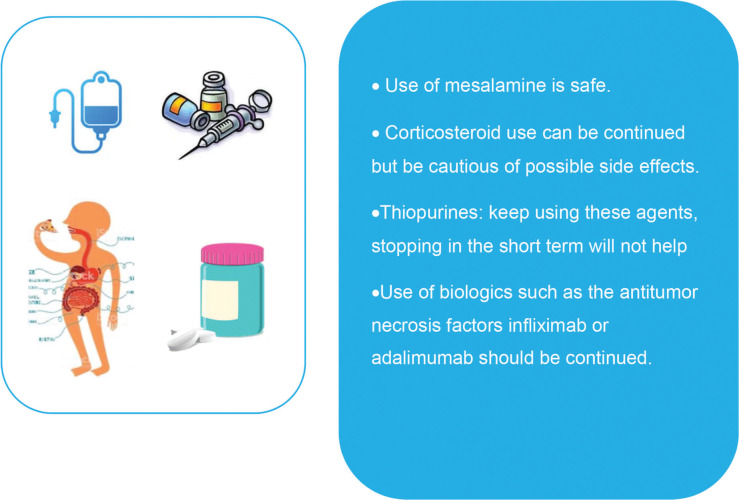
Recommendations on medications in patients with pediatric IBD during the pandemic COVID-19. Modified by Danese et al. (25).

In March 2019, recommendations provided by a group of experts from the International Organization of Inflammatory Bowel Disease (IOIBD) were described for adults (33). The IOIBD emphasizes, however, that the recommendations must be interpreted in the individual context of the patient with the assistance of their health professionals. They are not guidelines, and they can be updated as knowledge and the situation of the pandemic progresses (33). These recommendations may be discussed also in the context of pediatric IBD care (Figure 5).

**Figure 6:**
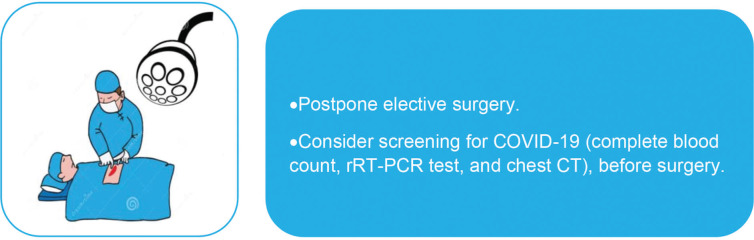
Recommendations on surgery in patients with pediatric IBD during the COVID-19 pandemic. Modified pandemic by Mao et al. (29).

Each of the medications used in the treatment of IBD was evaluated according to the increased risk for SARS-CoV-2 infection and also regarding its suspension in patients infected with SARS-CoV-2 with or without manifestation of the disease (Figure 6) (33).

### Recommendations for patients undergoing treatment with 5-aminosalicylic acid (5-ASA) and sulfasalazine infected with SARS-CoV-2 with or without manifestation of COVID-19 (33)**:**

- 5-ASA does not increase the risk of SARS-CoV-2 infection or COVID-19.
- Patients on therapy with 5-ASA should not reduce or discontinue the therapy dose to prevent SARS-CoV-2 infection.
- Patients on therapy with 5-ASA should not interrupt therapy if they are positive for SARS-CoV-2 with or without the manifestation of COVID-19.

### Recommendations for patients on treatment with budesonide infected with SARS-CoV-2 with or without manifestation of COVID-19 (33)**:**

- Budesonide does not increase the risk of SARS-CoV-2 infection or COVID-19.
- Patients on budesonide therapy should not reduce or interrupt the dose of therapy to prevent SARS-CoV-2 infection.
- It is uncertain whether patients using budesonide should discontinue treatment if they are positive for SARS-CoV-2 with or without COVID-19.

### Recommendations for patients on treatment with corticosteroids infected with SARS-CoV-2 with or without manifestation of COVID19 (33)**:**

- Prednisone (≥20 mg/d) increases the risk of infection with SARS-CoV-2 and COVID-19.
- Patients being treated with prednisone (≥20 mg/d) should reduce the dose of the therapy, if possible, to prevent infection by SARS-CoV-2.
- Patients on prednisone therapy (≥20 mg/d) should discontinue therapy (staggered reduction) if they are positive for SARS-CoV-2 or develop COVID-19.

### Recommendations for patients undergoing treatment with azathioprine, 6-mercaptopurine (6-MP), or methotrexate infected with SARS-CoV-2 with or without manifestation of COVID-19 (33):

- It is uncertain whether azathioprine, 6-MP, or methotrexate increases the risk of infection with SARS-CoV-2 or COVID-19.
- Patients using azathioprine, 6-MP, or methotrexate should not reduce the dose or discontinue therapy to prevent SARS-CoV-2 infection.
- Patients using azathioprine, 6-MP, or methotrexate should discontinue treatment if they are positive for SARS-CoV-2 or develop COVID-19.

### Recommendations for patients undergoing treatment with anti-TNF agents approved by the National Health Surveillance Agency for pediatric IBD (infliximab and adalimumab) infected with SARS-CoV-2 with or without the disease (COVID-19) (33):

- It is uncertain whether anti-TNF therapy increases the risk of infection with SARS-CoV-2 or COVID-19.
- Patients on anti-TNF therapy should not reduce the dose or discontinue therapy to prevent SARS-CoV-2 infection. According to ESPGHAN recommendations, the exchange of infliximab for adalimumab should not be encouraged due to the risk of relapse.
- It is unknown whether patients on anti-TNF therapy should discontinue therapy if they only show positive laboratory results for SARS-CoV-2 but have no clinical manifestation of the disease.
- Patients on anti-TNF therapy should stop the immunobiological if they develop COVID-19.

Patients undergoing combination therapy must be reexamined individually. However, currently the interruption of combination therapy is not a general recommendation for all cases in the pandemic.

In children, at the moment, it does not seem generally appropriate to recommend the interruption of immunosuppressants in the treatment of patients with pediatric IBD, except in cases of positive SARS-CoV-2 or COVID-19.

During the COVID-19 pandemic, screening for SARS-CoV-2 is recommended before starting immunosuppressive medications, to avoid immunosuppression in infected patients.

### 5. How to manage pediatric IBD patients who need surgery during the COVID-19 pandemic?

**Figure 7:**
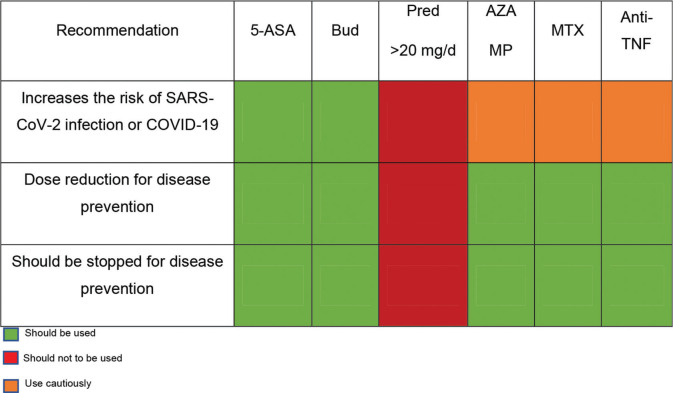
Adapted from the recommendations of the International Organization of Inflammatory Bowel Disease (IOIBD) (33).

Elective surgeries must be postponed during the COVID-19 pandemic. It is necessary to analyze each patient individually, to avoid exposing the patient to health risks, and therefore, in cases of unfavorable evolution, surgical indication may be necessary (Figure 7).

The Brazilian College of Surgeons published a planning strategies consensus for urgent/emergency surgeries during the SARS-CoV-2 pandemic period (34).

It is ideally recommended to screen for COVID-19 in cases of patients requiring emergency surgery.

### 6. When to restart immunosuppression of patients in the postoperative period of IBD during the pandemic?

There are no published data on prophylaxis for surgical recurrence during the COVID-19 pandemic. In general, the choice of medication for prophylaxis of surgical recurrence, regardless of the pandemic, should be based on patient risk assessment, but it is always important to ensure that the patient is not infected with SARS-CoV-2 (35).

## CONCLUSION

In summary, based on the available data, children have a predominantly benign course of COVID-19 with IBD, with mild symptoms and almost no reported mortality.

Children may play a major role in community-based viral transmission, especially in the home environment. In infants, it is necessary to be careful while changing diapers now that the isolation of the virus in stool samples from patients with COVID-19 has proven that SARS-CoV-2 can spread through feces.

Children with IBD, with and without immunosuppressive and biological therapy, appear to be at no greater risk of contracting SARS-CoV-2 infection compared to the general population.

It is considered that IBD in children tends to be more extensive and severe than in adults with a consistently higher need for immunomodulators and biological agents for them to remain in remission.

It may be cautiously suggested that there are currently no signs indicating a worsening of the COVID-19 course due to IBD-related treatment. On the other hand, the risk of inadequate management of IBD caused by the fear of the virus can have a significant impact on the health of IBD patients, as indicated by the increase in relapses in those who have not followed treatment regularly and properly. These temporary findings may be adjusted in the future based on emerging data from COVID-19 in children with IBD.

## AUTHOR CONTRIBUTIONS

Rodrigues M and Fragoso RP conceived the study, and were responsible for the data curation, formal analysis, investigation, methodology, supervision, and manuscript drafting, editing, and review.
